# Supplementary figures and images for: Skeletal myotubes expressing ALS mutant SOD1 induce pathogenic changes, impair mitochondrial axonal transport, and trigger motoneuron death
Source: Mol Med. 2024 Oct 25;30:185. doi: 10.1186/s10020-024-00942-4 (PMC11505737; doi:10.1186/s10020-024-00942-4)

# Supplemental figure 1

A

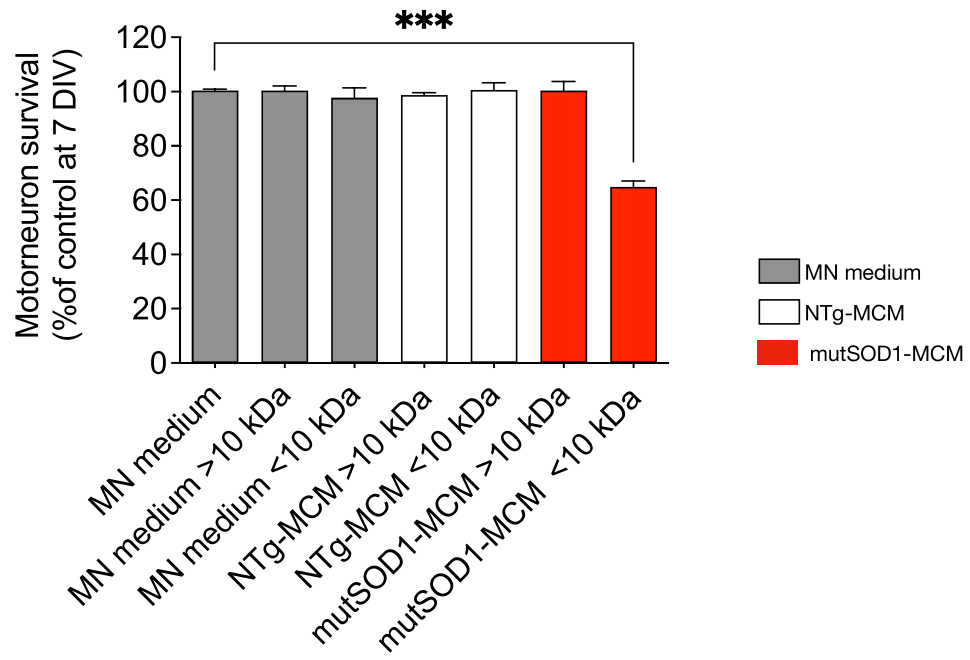

B

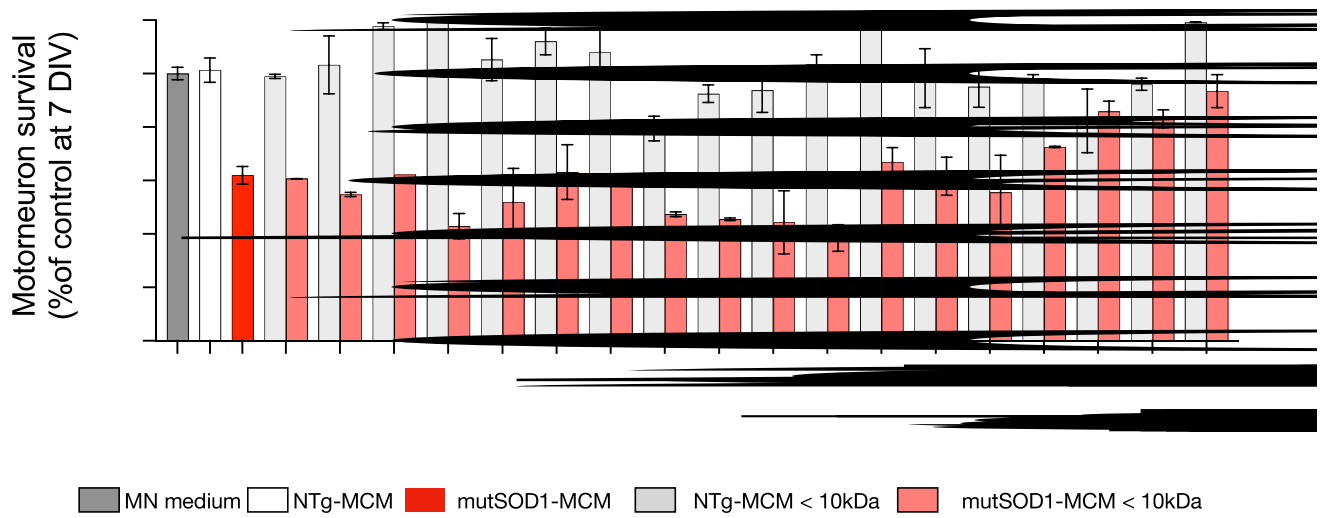

Supplement: Supplementary file 1 — Supplementary material 1: Figure S1. Small molecules <10 kDa from mutSOD1-MCM are responsible for MN death. A, MN survival graph (SMI32+/MAP2+ cells as a percentage of total MAP2+ neurons) after treatment for 3 days with MN medium, NTg-MCM, MCM-mutSOD1, and both NTg-MCM and MCM-mutSOD1 when passed through >10 kDa and <10 kDa filters. Values represent the mean ± s.e.m of at least three independent experiments and analyzed by one-way ANOVA (*** P <0.001). B, MN survival graph (SMI32+/MAP2+) after treatment for 3 days with MN medium, NTg-MCM, MCM-mutSOD1, and both <10 kDa filtered NTg-MCM and MCM-mutSOD1 when further passed through a Size Exclusion Chromatography (SEC). Results from 2 independent three independent experiments. [file 10020_2024_942_MOESM1_ESM.pdf]
